# Immune inactivation by VISTA predicts clinical outcome and therapeutic benefit in muscle-invasive bladder cancer

**DOI:** 10.1186/s12885-023-11157-x

**Published:** 2023-07-14

**Authors:** Wandi Li, Zhaopei Liu, Kaifeng Jin, Fei Shao, Han Zeng, Yiwei Wang, Yu Zhu, Le Xu, Zewei Wang, Yuan Chang, Weijuan Zhang

**Affiliations:** 1 Department of Immunology, School of Basic Medical Sciences, Fudan University, Shanghai, 200032 China; 2 Department of Biochemistry and Molecular Biology, NHC Key Laboratory of Glycoconjugate Research, School of Basic Medical Sciences, Fudan University, Shanghai, China; 3 Department of Urology, Fudan University Shanghai Cancer Center, Shanghai, 200032 China; 4 Department of Urology, Zhongshan Hospital, Fudan University, Shanghai, 200032 China; 5 Department of Oncology, Shanghai General Hospital, Shanghai Jiao Tong University School of Medicine, Shanghai, China; 6 Department of Urology, Shanghai Ninth People’s Hospital, Shanghai Jiao Tong University School of Medicine, Shanghai, China; 7 Department of Urology, Ruijin Hospital, Shanghai Jiao Tong University School of Medicine, Shanghai, China

**Keywords:** V domain immunoglobulin suppressor of T cell activation, Muscle-invasive bladder cancer, Adjuvant chemotherapy, Immunotherapy, Tumor microenvironment

## Abstract

**Background:**

V domain Immunoglobulin suppressor of T cell activation (VISTA) has been proved to be a novel immune checkpoint molecule that positively regulates T cell infiltration in several malignancies. However, the clinical impact of VISTA on muscle-invasive bladder cancer (MIBC) patients remains relatively obscure.

**Methods:**

This study enrolled 135 MIBC patients from Zhongshan Hospital (ZSHS) and 391 patients from The Cancer Genome Atlas (TCGA) to examine the VISTA expression and immune contexture based on immunohistochemistry (IHC) staining and CIBERSORT algorithm. Additionally, IMvigor210 Cohort included 195 bladder-derived urothelial carcinoma patients to evaluate the efficacy of immunotherapy. Kaplan-Meier curve and Cox regression analyses were conducted to assess clinical outcomes.

**Results:**

MIBC patients with high VISTA^+^ immune cells (ICs) possessed poor overall survival and inferior therapeutic responsiveness to adjuvant chemotherapy (ACT), but superior responsiveness to PD-L1 inhibitor. VISTA^+^ ICs infiltration shaped an immunoevasive context featured by regulatory T cells (Tregs), M2 macrophages, mast cells and exhausted CD8^+^ T cells infiltration, with increased interleukin 10 (IL-10), transforming growth factor-β (TGF-β) and interferon-γ (IFN-γ), but also elevated T-cell immunoglobulin mucin-3 (TIM-3), lymphocyte activation gene 3 (LAG-3) and T-cell immunoglobulin and ITIM domain (TIGIT), which was also mainly presented in basal-squamous and luminal-infiltrated subtypes of MIBC.

**Conclusion:**

VISTA^+^ ICs infiltration could be an independent predictor to identify poor prognosis and therapeutic responses (PD-L1 blockade and ACT) in MIBC patients, which was associated with immunoevasive contexture. The novel immune checkpoint VISTA might be utilized as a candidate treatment biomarker in MIBC patients.

**Supplementary Information:**

The online version contains supplementary material available at 10.1186/s12885-023-11157-x.

## Background

Bladder cancer is the most widespread and complex urinary tract malignancy all over the world with high-risk mortality, 25% of which are diagnosed as muscle-invasive bladder cancer (MIBC) [1]. MIBC is a more aggressive stage with unfavorable prognosis [2]. Cisplatin-based adjuvant chemotherapy (ACT) after radical cystectomy (RC) is commonly considered as the mainstay of current treatment for MIBC [3]. Unfortunately, the therapeutic efficacy is still far from satisfactory for advanced patients with metastases [4]. With the approval of nivolumab (anti-PD-1) from the US Food and Drug Administration (FDA) in MIBC [5], immune checkpoint inhibitors (ICIs) have shown a resounding success among cancer therapeutic strategies, of which regulatory molecules in B7 family are promising targets [6, 7]. Nevertheless, existing biomarker failed to cover all the responders, which could be partly attributed to the heterogeneity of tumor microenvironment (TME) [8, 9]. Consequently, there is an urgent need to seek predictive biomarkers for existing treatment and novel therapeutic paradigm for non-responders.

V domain immunoglobulin suppressor of T cell activation (VISTA), also known as VSIR, PD-1H, C10orf54, Dies1, DD1α and Gi24, belongs to the immune checkpoint proteins of B7 family, which is homologous to PD-L1 [10, 11]. VISTA serves a crucial function in regulating the immune system, preserving the stability of the intracellular environment [12]. Nonetheless, cancer cells could exploit VISTA to evade immune defenses in MIBC. Therefore, the development of alternative treatment strategies to the evasion mechanisms of MIBC are imperative to maximize anti-tumor efficacy. Investigating how VISTA interact between immune cells (ICs) and tumor cells (TCs) could facilitate the development of personalized therapies for MIBC patients.

VISTA has provided prognostic value and demonstrated the potential as an immunotherapy target for the patients. Elevated VISTA was associated with unfavorable outcomes across multiple malignancies [13–15]. Paradoxically, VISTA in TCs, but not in ICs, was significantly associated with prolonged survival in pancreatic cancer, hepatocellular carcinoma and high-grade serous ovarian cancer [16–18]. Furthermore, previous studies suggested that VISTA-positive ICs correlated with shorter recurrence-free in non-muscle-invasive bladder cancer (NMIBC) [19], however, its predictive value in MIBC remains unclear. The study of VISTA in MIBC could contribute to improved risk stratification and personalized treatment options.

In this work, we found that VISTA^+^ ICs infiltration indicated miserable clinical outcomes and poor responsiveness to ACT. Nonetheless, VISTA^+^ ICs infiltration in turn possessed a superior responsiveness to ICIs. In-depth transcriptomic and histological studies uncovered the immunosuppressive TME and presented basal-squamous and luminal-infiltrated subtype in VISTA^+^ ICs high subgroup, which might account for prognosis and therapeutic response. Our study unraveled the potential of VISTA as a novel candidate biomarker for MIBC patients.

## Methods

### Study patients

This study enrolled three independent cohorts, Zhongshan Hospital (ZSHS) Cohort, The Cancer Genome Atlas (TCGA) Cohort and IMvigor210 Cohort. The selecting procedure of studying cohorts was summarized in Supplementary Figure 1. The clinicopathological characteristics of patients were listed in Supplementary Table 1-3.

For ZSHS Cohort, 215 patients who received radical cystectomy (RC) at Zhongshan Hospital from 2002 to 2014 were followed up regularly till July 2016. 80 patients were ruled out due to the exclusion criteria: (1) postoperative histopathological diagnosis of non-urothelial carcinoma (UC) (*n*=13) or NMIBC (*n*=60), (2) unavailable in paraffin-embedded tumor tissues (*n*=7). 135 cases were enrolled in this study ultimately. The pathological type of all these patients was pure UC. Among them, 65 patients received ACT for at least one therapeutic cycle. The follow-up protocol was instructed by European Association of Urology guidelines for MIBC. Overall survival (OS) was calculated as the time from the date of RC to the date of death from all causes, or to the last follow-up.

TCGA Cohort enrolled 412 bladder cancer patients whose clinical information was downloaded from http://www.cbioportal.org/ in July 2021, 21 patients were excluded because of: (1) missing survival time (*n*=3), (2) unaccessible sequencing data (*n*=4), (3) accepted neoadjuvant chemotherapy (*n*=10), (4) postoperative histopathological diagnosis of NMIBC (*n*=4). 391 patients were enrolled according to the inclusion criteria of TCGA Cohort. Among them, 335 patients were classified as pure UC histology. 51 patients had UC with variant histology. 5 additional tumors were included: 1 bladder adenocarcinoma, 1 squamous cell carcinoma of non-bladder origin, and 3 pure squamous cell bladder carcinomas.

IMvigor210 trial originated from 348 metastatic UC patients treated with anti-PD-L1 agent atezolizumab [20]. In this study, we enrolled 195 bladder-derived urothelial cancer patients as IMvigor210 Cohort. The clinical and RNA-seq data were obtained through http://research-pub.gene.com/IMvigor210CoreBiologies. All of these patients had UC that had either been histologically or cytologically proven to be locally advanced or metastatic, including metastasis from the renal pelvis, ureter, urinary bladder, or urethra.

### Immunohistochemistry

All the bladder cancer tissues were obtained from the bladder specimens of 215 patients in ZSHS Cohort, which were subsequently formalin-fixed and paraffin-embedded. Before the construction of tissue microarray (TMA), 4 μm-thick sections were sliced from each tissue block. All samples were reviewed histologically by hematoxylin and eosin staining, and representative areas were marked on the paraffin blocks away from necrotic and hemorrhagic materials. Besides, each section of TMA was stained at the same time to guarantee an objective comparison between different samples. The protocol of immunohistochemistry (IHC) was executed as previously described [21]. Antibodies for VISTA and other molecules were provided in Supplementary Table 4.

### Assay methods

TMA slides were scanned under high-power magnification filed (HPF, 200 magnification) on NanoZoomer-XR (Hamamatsu) and scored by means of software ImageJ. All stained tissues were counted independently by two pathologists who were blind to the clinical and follow-up data. For the accurate purpose of statistical evaluation, we adopted as the mean value of cells infiltration in three representative fields (HPF, × 200 magnification). Consistent to previous reports [13, 14, 16, 17], we adopted a method of counting VISTA expressed in ICs or TCs separately in the immunohistochemical scoring of MIBC patients in ZSHS Cohort. The cut-off value of VISTA^+^ ICs in ZSHS Cohort was 37 cells/HPF, which determined by the R package survMisc (https://CRAN.R-project.org/package=survMisc). Whether VISTA expressed in TCs could be divided into positive and negative subgroup. Patients were dichotomized into VISTA^+^ ICs signature low and high subgroups in TCGA and IMvigor210 Cohorts also based on R package survMisc to identify the optimal cut-off values.

### RNA-seq and data processing

RNA-seq data of both TCGA Cohort and IMvigor210 Cohort were normalized by the formula log_2_(FPKM+1) before analysis. Based on the immune cell subsets expressing VISTA, ligand-receptor relationship and involved immune regulation process of VISTA, we used the average mean of the mRNA expression of *VSIR, IL6, IL10, IGSF11, SELPLG, VSIG8, ESAM, CD45* to constitute VISTA^+^ ICs signature [10, 22]. The infiltration of 22 ICs in TCGA Cohort was calculated by CIBERSORT algorithm, of which the sum was considered as the absolute score of each case. The involved signatures for gene set enrichment analysis (GSEA) were defined from previous studies or downloaded from https://gsea-msigdb.org and showed in Supplementary Table 5.

### Genomic analysis

Tumor mutation burden (TMB) is broadly identified as the number of somatic mutations per megabase of interrogated genome sequence (mut/Mb) [23]. Generally, TMB≥10 mut/Mb is identified as TMB^hi^ [24]. Gene alterations involved in signaling pathways were used to describe the genome pattern, which incorporated mutations and copy number variation (CNV) [25, 26]. The types of mutation included nonsense, missense, splice site, in frame deletion, multi hit, frame shift insertion and frame shift deletion. The types of CNV included deletions and amplifications.

### Statistical analysis

The relationship of VISTA^+^ ICs infiltration with patients’ clinicopathological parameters were conducted by Chi-square test. Analyses of the different cells infiltration between subgroups were dealt with Student’s t test. Overall survival (OS) was calculated from the date of operation until the date of death or last follow-up and disease-free survival (DFS) was calculated from the date of operation until the date of first recurrence or last follow-up. Kaplan-Meier curves for OS and DFS was evaluated by log-rank tests. Univariate and multivariate analyses were detected by constructing cox proportional hazard regression models. Gene Set enrichment analysis (GSEA) performed by three clusters of signaling signatures to identify the enrichment of exhausted CD8^+^ T cells in MIBC. In our study, all of data in the figure were shown as means ± SDs. *P* value of less than 0.05 was considered statistically significant. IBM SPSS Statistics 25.0 was utilized for all of the statistical analyses. Figures were visualized using MedCalc Statistical Software version 15.6.1, Graph Pad Prism Software version 7.0.1 and R software version 4.0.3.

## Results

### VISTA^+^ ICs infiltration indicates inferior survival outcomes in MIBC patients

**Fig. 1:**
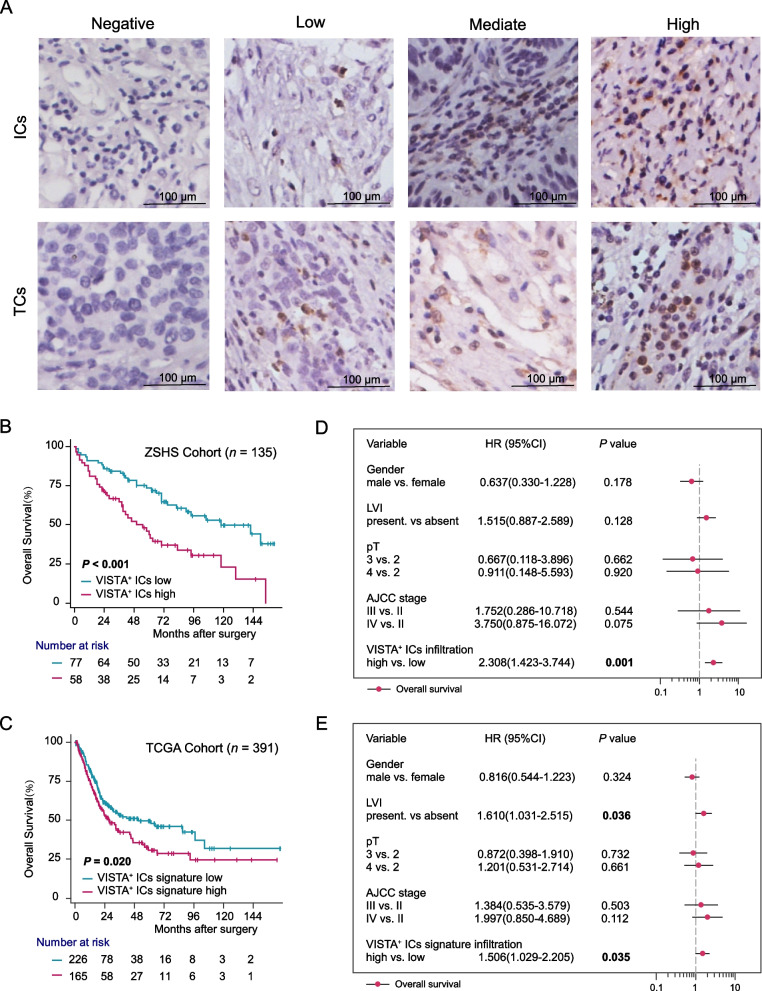
Prognostic significance of VISTA^+^ ICs infiltration in MIBC patients. Representative IHC images (200x magnification) of VISTA expression, including negative, low, mediate and high in ICs and TCs infiltration, respectively (**A**). Kaplan-Meier curves for OS in ZSHS Cohort (**B**) and TCGA Cohort (**C**) according to VISTA^+^ ICs infiltration. Data were analyzed using log-rank test. Multivariate cox analysis of OS was conducted on the basis of clinicopathologic characteristics and VISTA^+^ ICs infiltration in ZSHS Cohort (**D**) and TCGA Cohort (**E**). ICs, immune cells; TCs, tumor cells; OS, overall survival; HR, hazard radio; CI, confidence interval; LVI, lymphatic vesel invasion; AJCC, American joint committee on Cancer

Through immunostaining, we observed that VISTA expressed in both ICs and TCs, yet showed heterogeneous expression pattern (Fig. 1A). The number of VISTA^+^ ICs in every TMA ranged from 0 to 201 per HPF, however, VISTA was detected in TCs in only 36 samples among the 135 specimens. We further assessed the prognostic merit of VISTA^+^ ICs and TCs infiltration by Kaplan-Meier analysis in ZSHS Cohort. In contrast with VISTA^+^ TCs infiltration (OS: *p* = 0.842, DFS: *p* = 0.373, Supplementary Figure 2), VISTA^+^ ICs infiltration was relevant to adverse prognosis (*p* < 0.001, Fig. 1B). To verify this finding, we incorporated TCGA Cohort and found VISTA^+^ ICs signature high subgroup also had inferior OS (*p* =0.020, Fig. 1C). Therefore, we focused in VISTA^+^ IC infiltration in the following study. We further performed univariate (Supplementary Table 6-7) and multivariate (Fig. 1D, E, Supplementary Table 8) Cox regression analysis to evaluate predictive value of VISTA for clinical outcomes. After adjusting for gender, lymphatic vessel invasion (LVI), pT, American Joint Committee on Cancer (AJCC), we demonstrated that VISTA^+^ ICs infiltration could be regarded as an independent prognostic factor.

### VISTA^+^ ICs infiltration yields suboptimal adjuvant chemotherapeutic responsiveness in MIBC patients

**Fig. 2:**
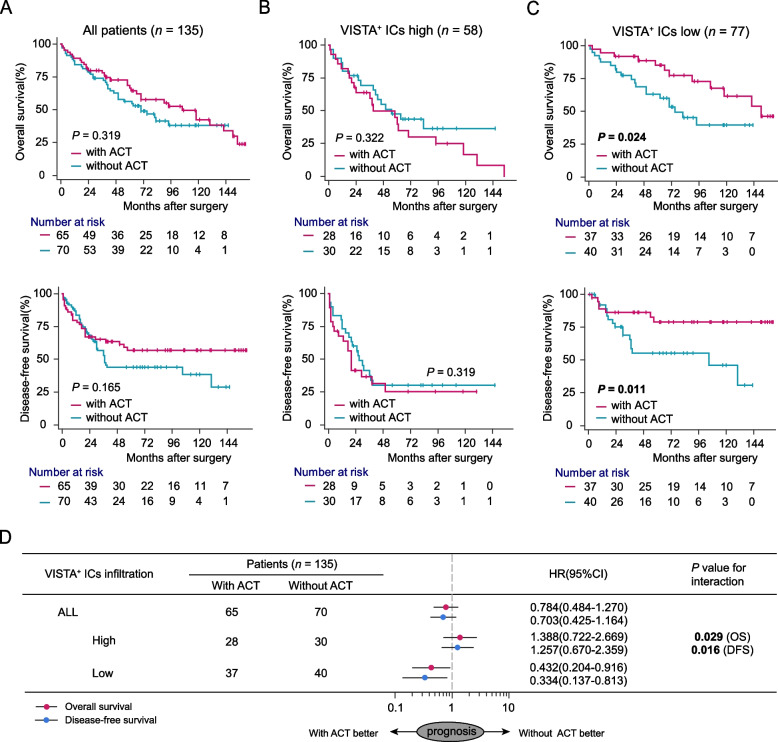
VISTA^+^ ICs infiltration predicts suboptimal responsiveness to adjuvant chemotherapy in MIBC patients. Kaplan-Meier curves for OS and DFS in all patients (**A**), VISTA^+^ ICs high subgroup (**B**) and VISTA^+^ ICs low subgroup (**C**) with or without ACT treatment.(**D** Cox regression analyses of OS and DFS for an interaction in patients with or without ACT according to VISTA^+^ ICs infiltration. OS, overall survival; DFS, disease-free survival; ICs, immune cells; ACT, adjuvant chemotherapy; HR, hazard radio; CI, confidence interval

All the MIBC patients in ZSHS Cohort failed to benefit from cisplatin-based ACT (OS: *p* = 0.319, DFS: *p* = 0.165, Fig. 2A). Herein, we further examined the potential impact of VISTA^+^ ICs infiltration on the effectiveness of ACT. Intriguingly, compared with VISTA^+^ ICs high subgroup (OS: *p* = 0.322, DFS: *p* = 0.319, Fig. 2B), VISTA^+^ ICs low subgroup had prolonged OS and DFS (OS: *p* = 0.024, DFS: *p* = 0.011, Fig. 2C). Subgroup interaction analysis further illustrated that patients with low VISTA^+^ ICs infiltration would possess more clinical benefits from ACT (OS: *P* = 0.029, DFS: *P* = 0.016 for interaction, Fig. 2D). Collectively, these results unveiled VISTA^+^ ICs high subgroup was associated with chemotherapeutic resistance to ACT in MIBC.

### VISTA^+^ ICs infiltration might guide the application of anti-PD-L1 therapy in MIBC patients

**Fig. 3:**
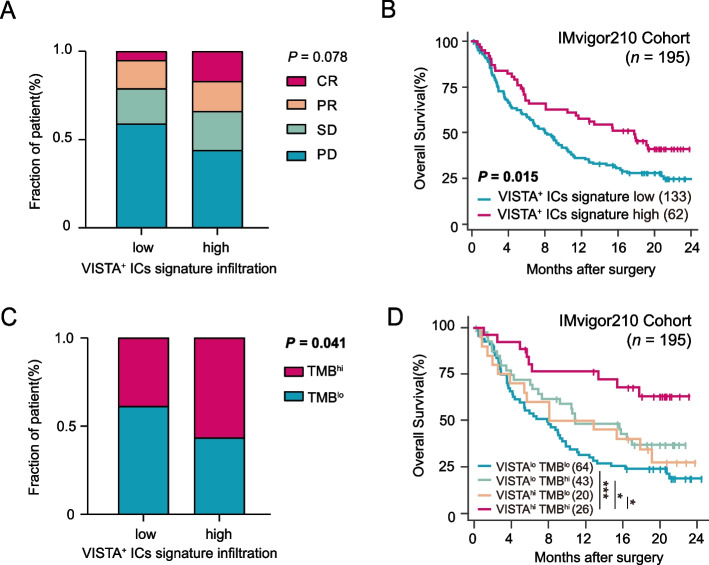
Predictive value of VISTA^+^ ICs infiltration for immunotherapy in IMvigor210 Cohort. **A** Fractions of objective response to atezolizumab between VISTA^+^ ICs signature low and high subgroup in IMvigor210 Cohort. Data were analyzed by Chi-square test. **B** Kaplan-Meier curves for OS in IMvigor210 Cohort according to VISTA^+^ ICs infiltration. Data were analyzed using log-rank test. **C** Fractions of TMB between VISTA^+^ ICs signature low and high subgroup in IMvigor210 Cohort. Data were analyzed by Chi-square test. **D** Kaplan-Meier curves of OS in IMvigor210 Cohort according to 4 subgroups divided by VISTA^+^ ICs infiltration and TMB. CR, complete response; PR, progressive response; SD, stable disease; PD, partial disease; OS, overall survival; TMB, tumor mutation burden

Besides ACT, ICIs have emerged as a remarkable approach for the treatment of cancer [7]. To research the predictive potential of VISTA^+^ ICs infiltration in ICIs treatment, we enrolled IMvigor210 Cohort in which patients treated with atezolizumab. Our finding suggested that patients with VISTA^+^ ICs infiltration had higher response rates (*p* = 0.078, Fig. 3A) and reflected significantly improved OS after atezolizumab application (*p* = 0.015, Fig. 3B). In addition, we examined the association of VISTA^+^ ICs infiltration and TMB to predict anti-PD-L1 immunotherapy responsiveness effectively [27]. Our studies reported that TMB^hi^ might reflect an increased potential for immunogenicity in VISTA^+^ ICs signature high subgroup (Fig. 3C). Considering that FDA has approved TMB as a valid predictor in treating metastatic UC [23], we classified MIBC patients into four subgroups according to VISTA^+^ ICs infiltration and TMB. Patients with VISTA^hi^TMB^hi^ feature had a superior prognosis compared with other groups (*p* < 0.001, Fig. 3D), which provided new ideas for individual precise immunotherapy in MIBC patients. In brief, our results illustrated that VISTA^+^ ICs infiltration had a better therapeutic response to PD-L1 inhibitor and could be a potential biomarker for ICIs treatment with MIBC patients.

### VISTA^+^ ICs infiltration shaped an immunosuppressive microenvironment in MIBC patient

**Fig. 4:**
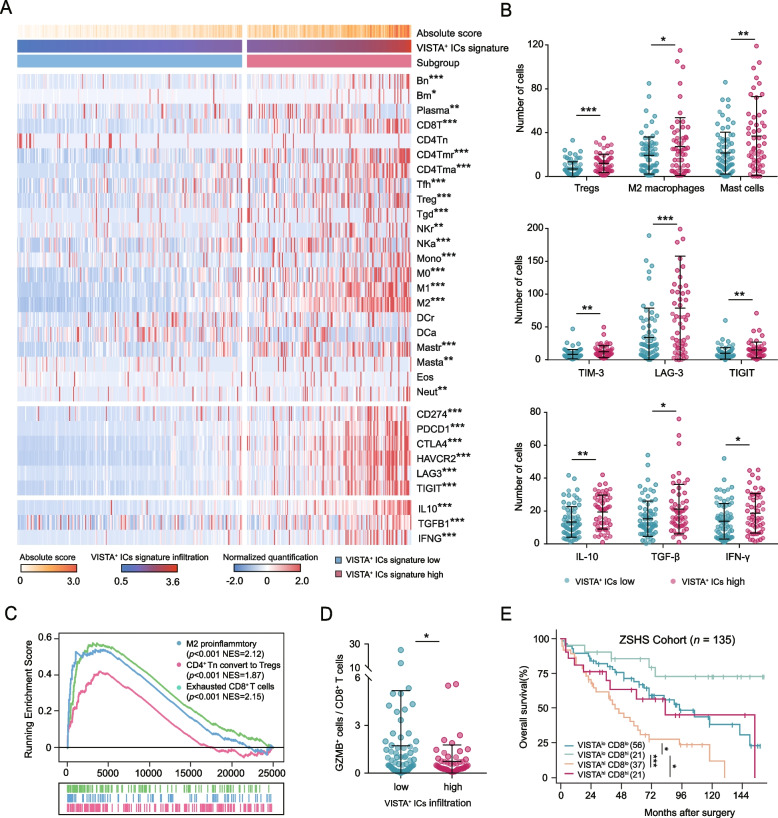
Identification of immunosuppressive microenvironment based on VISTA^+^ ICs infiltration in MIBC. **A** CIBERSORT quantification of 22 types of immune cells, immune checkpoints (CD274, PDCD1, CTLA4, HAVCR2, LAG3, TIGIT) and cytokines (IL10, TGFB1, IFNG) between VISTA^+^ ICs signature low and high subgroups in TCGA Cohort. **B** Immunohistochemistry analyzed the immune contexture of immune cells (Tregs, M2 macropahges, mast cells), immune checkpoints (TIM-3, LAG-3, TIGIT) and cytokines (IL10, TGF-β, IFN-γ) between VISTA^+^ ICs low and high subgroup in ZSHS Cohort. Data were analyzed by Student’s t test. **C** Gene set enrichment analysis to evaluate enrichment of published gene sets of Tregs, M2 macrophages, mast cells and exhausted CD8^+^ T cells, among genes ranked by their expression in VISTA^+^ ICs signature high versus low subgroup in TCGA Cohort. **D** Immunohistochemistry analyzed the ratio of GZMB^+^ cells to CD8^+^ T cells between VISTA^+^ ICs low and high subgroup in ZSHS Cohort. Data were analyzed by Student’s t test. **E** Kaplan-Meier curves for OS in ZSHS Cohort according to four subgroups divided by VISTA^+^ ICs and CD8^+^ T cells infiltration. *P < 0.05, **P < 0.01, ***P < 0.001 and ns *P* > 0.05. OS, overall survival; ICs, immune cells

To unravel the landscape of TME in VISTA^+^ ICs infiltration, we compared the immune infiltration across VISTA^+^ ICs subgroups. Despite the inflamed contexture, VISTA^+^ ICs infiltration indicated a suppressive TME with elevated expression of immune checkpoints and inhibitory cytokines in TCGA Cohort (Fig. 4A). We verified this finding in ZSHS Cohort and observed elevated pro-tumor cells in VISTA^+^ ICs high subgroup, including regulatory T cells (Tregs), M2 macrophages and mast cells (Tregs: *p* < 0.001, M2 macrophages: *p* = 0.033, Mast cells: *p* = 0.001, Fig. 4B, Supplementary Figure 3A). In addition, VISTA^+^ ICs infiltration was accompanied by the up-regulation expression of immune checkpoints like T-cell immunoglobulin mucin-3 (TIM-3), Lymphocyte activation gene 3 (LAG-3) and T-cell immunoglobulin and ITIM domain (TIGIT) (TIM-3: *p* = 0.007, LAG-3: *p* < 0.001, TIGIT: *p* = 0.006, Fig. 4B, Supplementary Figure 3B), and indicated the elevation of interleukin 10 (IL-10), transforming growth factor-β (TGF-β) and interferon-γ (IFN-γ) (IL-10: *p* < 0.001, TGF-β: *p* = 0.009, IFN-γ: *p* = 0.023, Fig. 4B). Notably, gene set enrichment analysis (GSEA) further manifested that Tregs and M2 macrophages-related signaling pathways were hyperactivated in VISTA^+^ ICs signature high subgroup (Fig. 4C).

Besides these inhibitory ICs infiltration, CD8^+^ T cells infiltration presented an exhausted phenotype and secreted decreased level of granzyme B (GZMB) in VISTA^+^ ICs infiltration (*p* = 0.037, Fig. 4C, D). Furthermore, stratification based on VISTA^+^ ICs and CD8^+^ T cells infiltration showed that patients with VISTA^lo^CD8^hi^ features learned more benefits than those with double high subgroup (*p* < 0.001, Fig. 4E), indicating that exhausted CD8^+^ T cells were closely related to VISTA^+^ ICs enrichment. Taken together, VISTA^+^ ICs infiltration was linked to immune enriched but suppressive TME and evaded immune control, which might account for the adverse prognosis with MIBC patients.

### Characterization of gene alterations based on VISTA^+^ ICs infiltration in MIBC patients

Chromosomal instability is critical for oncogenesis, of which continuous accumulation lead to intratumoral genetic heterogeneity [28]. Genomic alterations are especially frequent in bladder cancer, including somatic mutations and CNVs, and rearrangements, which are capable of determining oncogenesis, progression and sensitivity to therapy [29]. Herein, we profiled the distribution of gene alterations and molecular subtypes based on VISTA^+^ ICs infiltration in TCGA Cohort (Supplementary Figure 4). Patients with high VISTA^+^ ICs infiltration were mostly classified into basal-squamous and luminal-infiltrated subtypes. In addition, histone modification-related gene alterations were fewer enriched in VISTA^+^ ICs signature high subgroup (*p*=0.014, Supplementary Figure 4).

## Discussion

Nowadays, accurately predicting treatment responders is the main concern. Suitable biomarker for predicting treatment response will aid in identifying responders [29]. Numerous reviews suggested that increasing the antigenicity of cancer cells and inducing a more immunogenic microenvironment after conventional chemotherapy [30, 31]. However, our work reported that VISTA^+^ ICs infiltration was associated with inhibitory TME, which suppressed the further expanded immune effects. That’s the possible reason why VISTA^+^ ICs infiltration could be resistant to chemotherapy but favorable to derived more benefits from ICIs. TMB is a measure of the number of mutations in a cancer [32]. The more mutations (i.e., the higher the TMB) reflected the greater the chances that some neo-antigens would be immunogenic and enable T cell recognition [33, 34]. Recent research reported that TMB might not always correlate with ICIs responsiveness [35]. Developing precision therapies could improve clinical significance by stratification of patients who are resistant or sensitive to ICIs. Herein, we identified VISTA^hi^TMB^hi^ patients who possessed the outstanding responsiveness to ICIs based on integrating VISTA^+^ ICs infiltration stratification with TMB, which probably accounted for immune enriched and increased immunogenicity. The exact mechanisms still need to be further explored.

Accumulating evidences that the success of chemotherapy and immunotherapy could be partially attributed to the immune landscape of TME [36]. Our previous study showed that some specific subsets of ICs infiltration could influence the responsiveness of MIBC patients to chemotherapy [37–39]. Given the evasion of immune control from the standpoint of VISTA^+^ ICs infiltration, VISTA could promote the transformation of naive Foxp3^+^CD4^+^ T cells into adaptive Foxp3^+^ Tregs and induce polarized M2 macrophages [12, 40], Most Tregs and M2 macrophages generally released a high level of IL-10 and TGF-β, which exerted inhibitory influence on antigen-presenting capability of macrophages and T cells function [41]. It has been reported that terminally exhausted CD8^+^ T cells enrichment always accompanied with increasing immune checkpoints [37, 42], which could support our finding that CD8^+^ T cells played an exhausted role in VISTA^+^ ICs infiltration. Moreover, targeting VISTA has been shown to induce inflammatory mediators and modulate exhausted CD8^+^ T cells into antitumor effector T cells [43], suggesting VISTA blockade had great value in treating MIBC patients and could be a novel method of further clinical treatment.

Resent research concluded that IFN-γ could induce the upregulation of suppressive receptors on tumor cell and TAMs, such as PD-L1, and further upregulated the expression of VISTA to suppress the degree of antitumor immune responses [44, 45]. Similar to VISTA-positive ICs in NMIBC, VISTA^+^ ICs infiltration was associated with poor prognosis in MIBC [19], which indicated VISTA play a consistent and persistent role in bladder cancer disease progress. The expression of VISTA and PD-L1 in different ICs showed that they may have immunologic activities in NMIBC. Additionally, VISTA expression on antigen-presenting cells is distinct from the PD-1/PD-L1 in melanoma [46]. In murine, VISTA promoted tumor growth via regulation of T cells activation independent of PD-1/PD-L1 pathway [47]. In conclusion, VISTA demonstrated the valuable significance as a potential immunotherapy target, which aids the immune system in anti-tumor. Moreover, VISTA and PD-1 blockade could make more informed decisions regarding effective therapies and personalized treatment option for MIBC patients.

## Conclusion

We aimed to investigate clinical significance of VISTA in MIBC. Our study evaluated VISTA^+^ ICs infiltration as a candidate biomarker for predicting survival outcomes and therapeutic responsiveness. Specifically, VISTA^+^ IC high subgroup was refractory to ACT while favorable to PD-L1 inhibitor. Further we deciphered VISTA^+^ IC infiltration was associated with inhibitory tumor environment characterzied by Tregs, M2 macrophages, mast cells and exhausted CD8^+^ T cells infiltration, along with increased IL-10 and TGF-β, but also elevated immune checkpoint expression, such as TIM-3, LAG-3 and TIGIT, which mediated the immune evasion in MIBC. In brief, VISTA^+^ ICs infiltration could be as a promising biomarker for guiding precision medicine strategies for MIBC patients.

## Supplementary Information

**Additional file 1:** **Supplementary Figure 1.** The selecting procedure of studying cohorts. **Supplementary Figure 2.** Prognostic significance of VISTA^+^ TCs infiltration in MIBC patients. **Supplementary Figure 3.** Identification of immune cells and checkpoints based on VISTA^+^ ICs infiltration in ZSHS Cohort. **Supplementary Figure 4.** Somatic alterations in signaling pathways across VISTA^+^ ICs infiltration. **Supplementary Table 1.** Clinicopathological characteristics and relationship with VISTA^+^ cells infiltration in ZSHS cohort. **Supplementary Table 2.** Clinicopathological characteristics and relationship with VISTA^+^ ICs infiltration in TCGA cohort. **Supplementary Table 3.** Clinicopathological characteristics and relationship with VISTA^+^ ICs infiltration in IMvigor210 cohort. **Supplementary Table 4.** Immunohistochemistry antibodies and quantification. **Supplementary Table 5.** Specific gene signatures. **Supplementary Table 6.** Univariate analysis of clinicopathologic features and VISTA^+^ ICs/ VISTA^+^ TCs infiltration in ZSHS Cohort. **Supplementary Table 7.** Univariate analysis of clinicopathologic features and VISTA^+^ ICs signature infiltration in TCGA Cohort. **Supplementary Table 8.** Multivariate analysis of clinicopathologic features and VISTA^+^ TCs infiltration in ZSHS Cohort.

## Data Availability

All data generated and analyzed during this study are included in this published article and its supplementary information files. The clinical data and gene profiles of patients were downloaded from the public network. More datasets are available from the corresponding author Prof. Zhang on reasonable request.

## Abbreviations

VISTA: V domain immunoglobulin suppressor of T cell activation
ICs: Immune cells
TCs: Tumor cells
MIBC: Muscle-invasive bladder cancer
NMIBC: Non-muscle-invasive bladder cancer
UC: Urothelial carcinoma
ZSHS: Zhongshan Hospital
TCGA: The Cancer Genome Atlas
ACT: Adjuvant chemotherapy
ICIs: Immune checkpoint inhibitors
FDA: The US Food and Drug Administration
LVI: Lymphatic vessel invasion
AJCC: American Joint Committee on Cancer
TMB: Tumor mutation burden
CNV: Copy number variation
OS: Overall survival
DFS: Disease-free survival
IHC: Immunohistochemistry
TMA: Tissue microarray
HR: Hazard ratios
IQR: Interquartile range
RC: Radical cystectomy
TME: Tumor microenvironment
Tregs: Regulatory T cells
IFN-γ: Interferon-γ
IL-10: Interleukin 10
TGF-β: Transforming growth factor-β
GZMB: Granzyme B
PD-1: Programmed cell death protein 1
PD-L1: Programmed cell death 1 ligand 1
LAG-3: Lymphocyte activation gene 3
TIM-3: T-cell immunoglobulin mucin-3
TIGIT: T-cell immunoglobulin and ITIM domain
GSEA: Gene set enrichment analysis
