# Supplementary material for: Immune inactivation by VISTA predicts clinical outcome and therapeutic benefit in muscle-invasive bladder cancer
Source: BMC Cancer. 2023 Jul 14;23:661. doi: 10.1186/s12885-023-11157-x (PMC10347783; doi:10.1186/s12885-023-11157-x)
Supplement: Supplementary file 1 — Additional file 1: Supplementary Figure 1. The selecting procedure of studying cohorts. Supplementary Figure 2. Prognostic significance of VISTA+ TCs infiltration in MIBC patients. Supplementary Figure 3. Identification of immune cells and checkpoints based on VISTA+ ICs infiltration in ZSHS Cohort. Supplementary Figure 4. Somatic alterations in signaling pathways across VISTA+ ICs infiltration. Supplementary Table 1. Clinicopathological characteristics and relationship with VISTA+ cells infiltration in ZSHS cohort. Supplementary Table 2. Clinicopathological characteristics and relationship with VISTA+ ICs infiltration in TCGA cohort. Supplementary Table 3. Clinicopathological characteristics and relationship with VISTA+ ICs infiltration in IMvigor210 cohort. Supplementary Table 4. Immunohistochemistry antibodies and quantification. Supplementary Table 5. Specific gene signatures. Supplementary Table 6. Univariate analysis of clinicopathologic features and VISTA+ ICs/ VISTA+ TCs infiltration in ZSHS Cohort. Supplementary Table 7. Univariate analysis of clinicopathologic features and VISTA+ ICs signature infiltration in TCGA Cohort. Supplementary Table 8. Multivariate analysis of clinicopathologic features and VISTA+ TCs infiltration in ZSHS Cohort. [file 12885_2023_11157_MOESM1_ESM.docx]

**Supplementary Figure 1**

**Supplementary Figure 1 The selecting procedure of studying cohorts.**


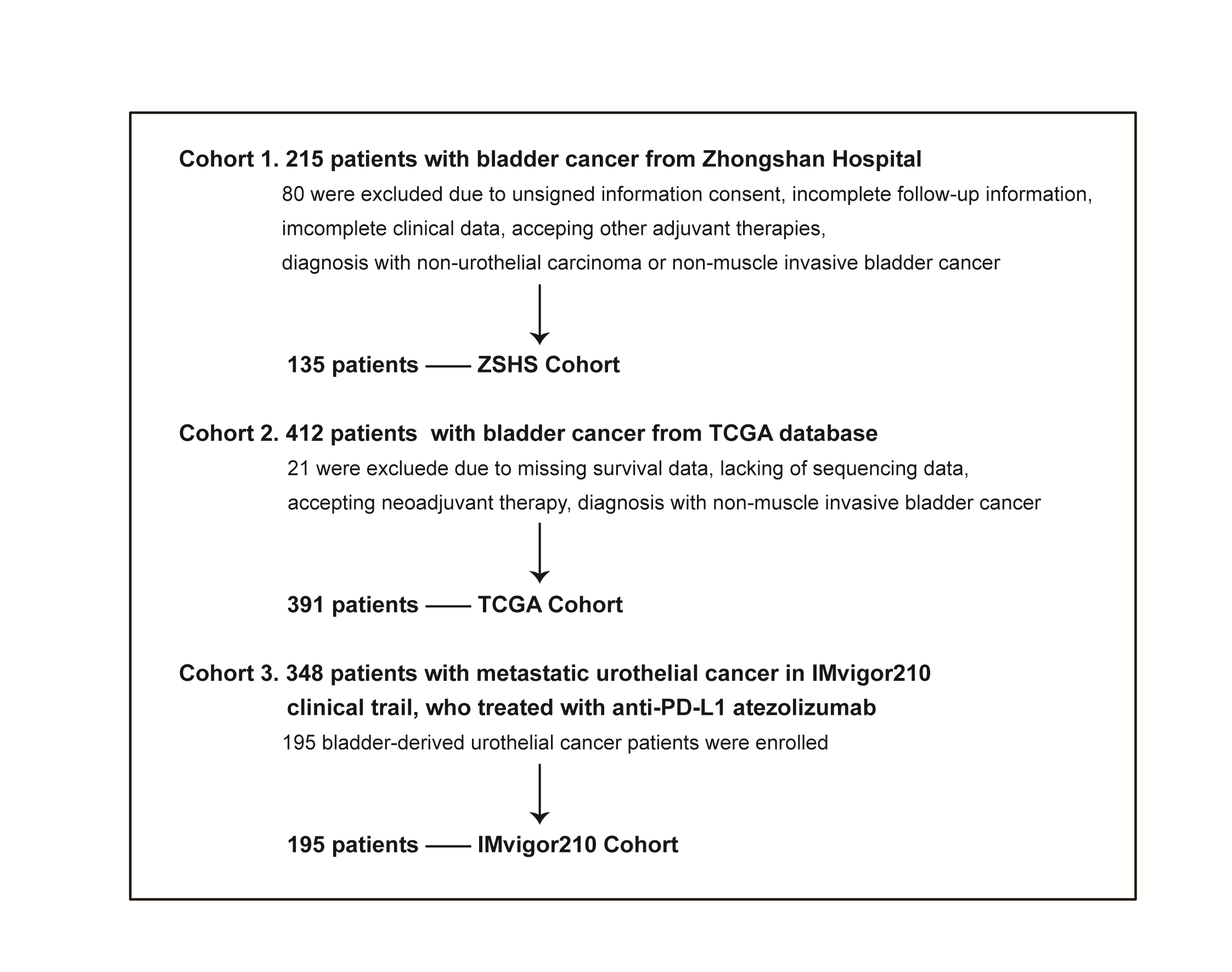


ZSHS Cohort included 215 consecutive patients who underwent radical cystectomy at Zhongshan Hospital of Fudan University in Shanghai, China, from 2002 to 2014, and were followed up regularly till July 2016, 80 of whom were ruled out. TCGA Cohort enrolled 412 bladder cancer patients whose clinical information was downloaded from <http://www.cbioportal.org/> in July 2021, 21 patients were excluded. IMvigor210 trial originated from 348 metastatic UC patients treated with anti-PD-L1 agent atezolizumab, the clinical and RNA-seq data were obatained through <http://research-pub.gene.com/IMvigor210CoreBiologies>. In this study, we enrolled 195 bladder-derived urothelial cancer patients as IMvigor210 Cohort.

**Supplementary Figure 2**


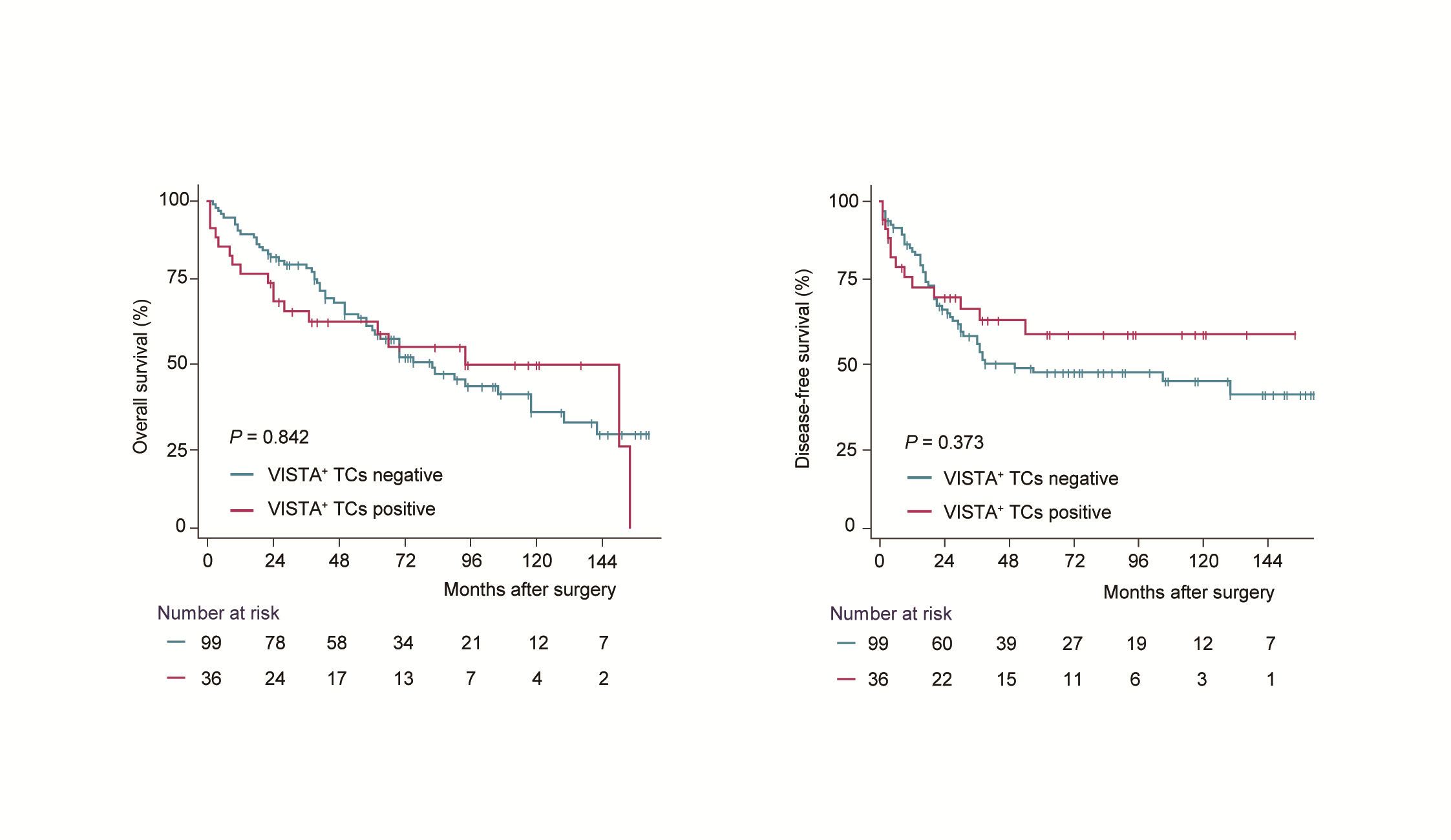


**Supplementary Figure 2 Prognostic significance of VISTA^+^ TCs infiltration in MIBC patients.**

Kaplan-Meier curves for OS and DFS in ZSHS Cohort according to VISTA^+^ TCs infiltration. Data were analyzed using log-rank test. OS, overall survival; DFS, disease-free survival; ICs, immune cells.

**Supplementary Figure 3**


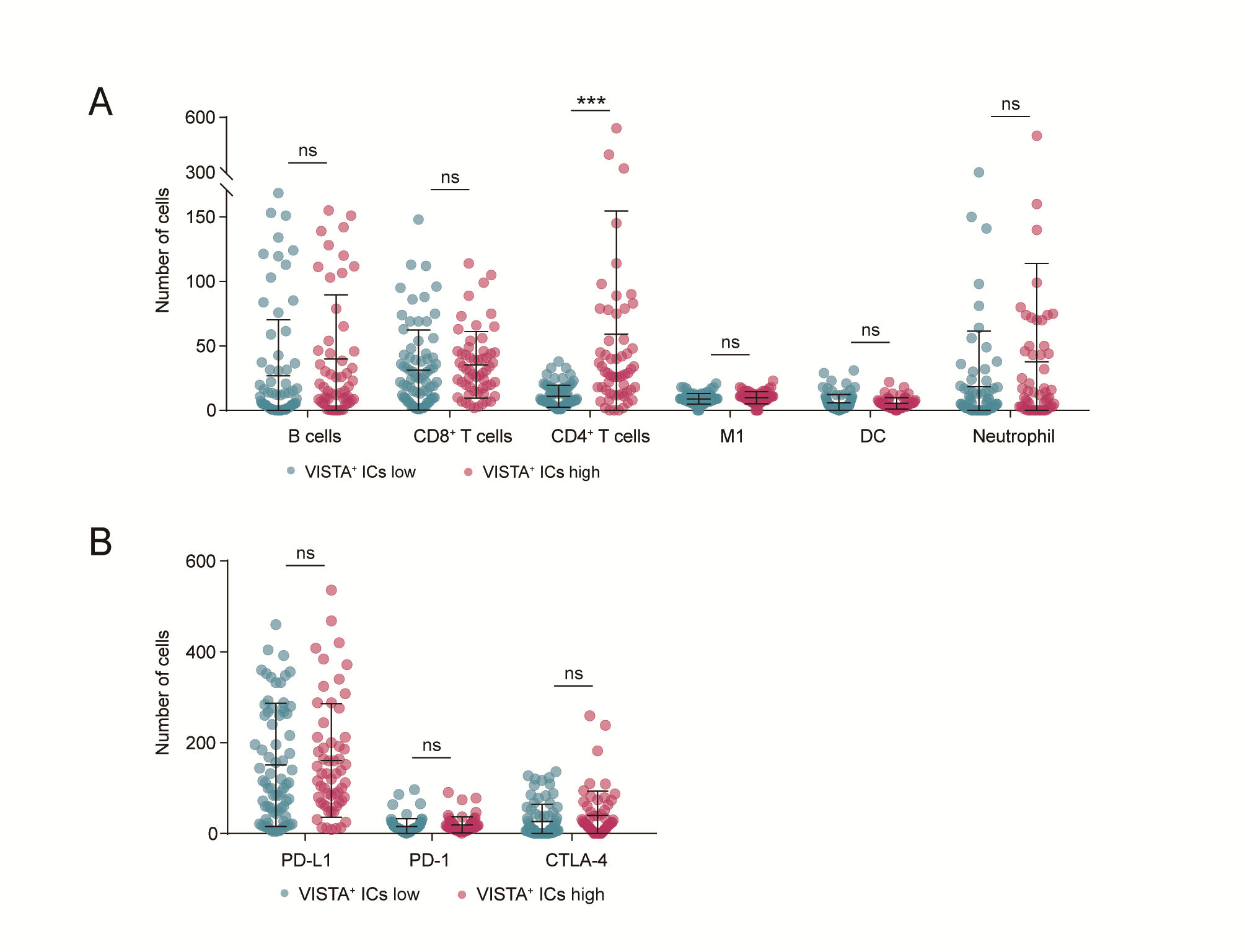


**Supplementary Figure 3 Identification of immune cells and checkpoints based on VISTA^+^ ICs infiltration in ZSHS Cohort.**

(A) Immunohistochemistry analyzed the immune contexture of immune cells (B cells CD8^+^ T cells, CD4^+^ T cells, M1 macrophages, DC, neutrophil), immune checkpoints (PD-L1, PD-1, CTLA-4) between VISTA^+^ ICs low and high subgroup in ZSHS Cohort. Data were analyzed by Student’s t test. **P* < 0.05, ***P* < 0.01, ****P* < 0.001 and ns *P* > 0.05.

**Supplementary Figure 4**

**
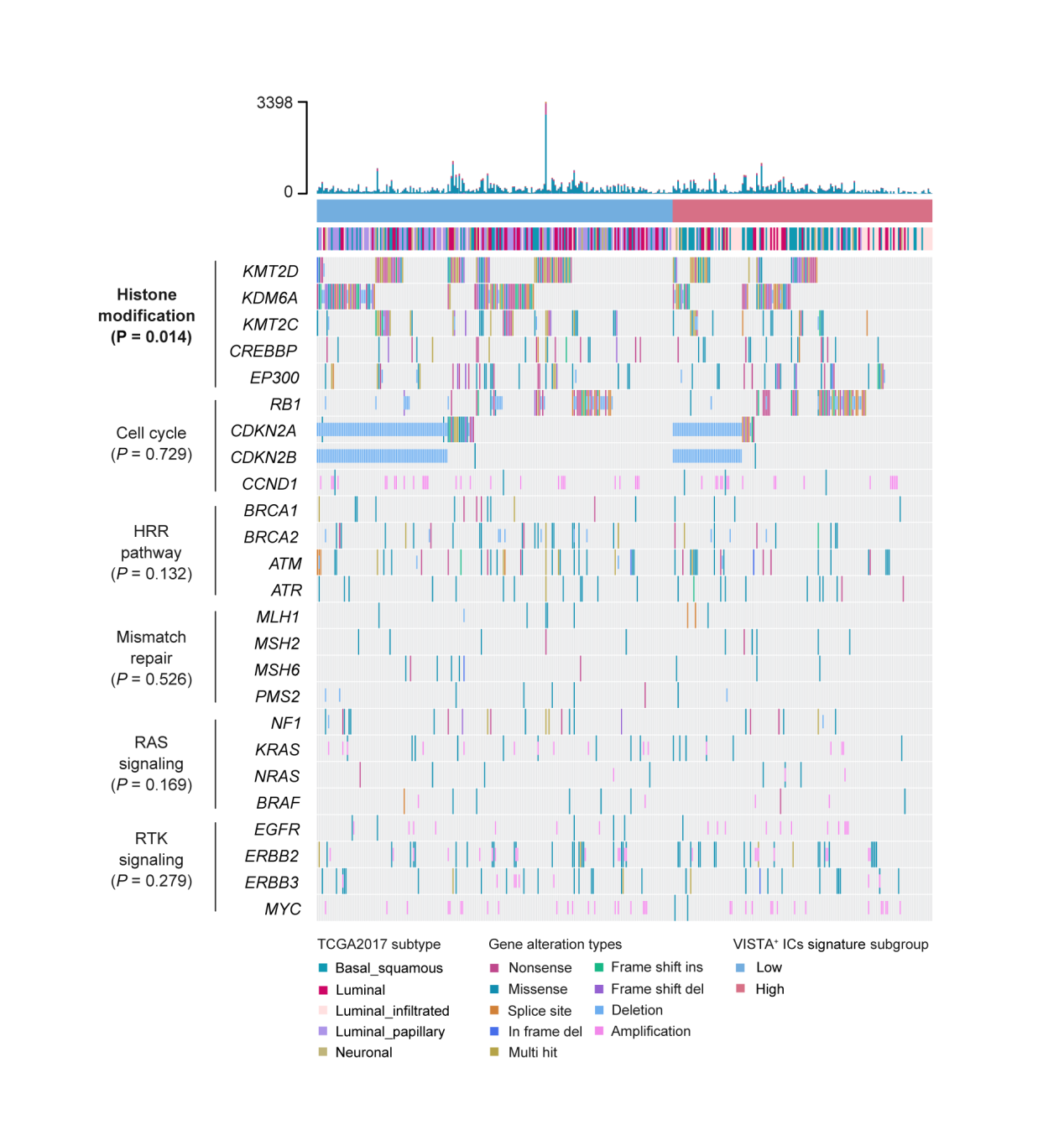
**

**Supplementary Figure 4 Somatic alterations in signaling pathways across VISTA^+^ ICs infiltration**

The OncoPrint was constructed to analysis different types of somatic alterations, including mutations and copy number variations between VISTA^+^ ICs signature low and high subgroup in TCGA Cohort. Colors indicate different somatic alteration types, shown at bottom. Data were analyzed by Chi-square test. HR, hazard radio; CI, confidence interval; WT, wild type; GA, gene alteration

| **Supplementary Table 1 Clinicopathological characteristics and relationship with VISTA^+^ cells infiltration in ZSHS cohort.** | | | | | | | | | | | | | | | |  |  |
| --- | --- | --- | --- | --- | --- | --- | --- | --- | --- | --- | --- | --- | --- | --- | --- | --- | --- |
|  | | **ZSHS cohort ( *n* = 135 )** | | | | | | | | | | | | | |  |  |
| **Characteristics** | | **Patients** | | | **VISTA^+^ ICs** | | | |  | **Patients** | | **VISTA^+^ TCs** | | |  | | |
|  |  | **No.** | | **Low** | | **High** | ***P**** | | **No.** | | **Negative** | | **Positive** | ***P**** | | |  |
|  |  |  | | **(**<**37 cells/HPF)** | | **(**≥**37 cells/HPF)** |  |  |  | | **(**<**1 cells/HPF)** | | **(**≥**1 cells/HPF)** |  |  |  |  |
| **Total** | | 135 | | 77 | | 58 |  | | 135 | | 99 | | 36 |  | | |  |
| **Age** | |  | |  | |  | 0.239 | |  | |  | |  | 0.754 | | |  |
| Median (IQR) | | 62(56-72) | | 62(54-70) | | 58(64-72) |  | | 62(56-72) | | 62(56-71) | | 64(55-73) |  | | |  |
| **Gender** | |  | |  | |  | 0.327 | |  | |  | |  | 0.138 | | |  |
|  | Male | 112 | | 66 | | 46 |  | | 112 | | 85 | | 27 |  | | |  |
|  | Female | 23 | | 11 | | 12 |  | | 23 | | 14 | | 9 |  | | |  |
| **LVI** | |  | |  | |  | 0.270 | |  | |  | |  | 0.100 | | |  |
|  | Absent | 49 | | 31 | | 18 |  | | 49 | | 40 | | 9 |  | | |  |
|  | Present | 86 | | 46 | | 40 |  | | 86 | | 59 | | 27 |  | | |  |
| **pT stage** | |  | |  | |  | 0.945 | |  | |  | |  | 0.494 | | |  |
|  | pT2 | 86 | | 49 | | 37 |  | | 86 | | 64 | | 22 |  | | |  |
|  | pT3 | 29 | | 16 | | 13 |  | | 29 | | 19 | | 10 |  | | |  |
|  | pT4 | 20 | | 12 | | 8 |  | | 20 | | 16 | | 4 |  | | |  |
| **pN stage** | |  | |  | |  | 0.678 | |  | |  | |  | 0.079 | | |  |
|  | pN0 | 127 | | 73 | | 54 |  | | 127 | | 91 | | 36 |  | | |  |
|  | pN+ | 8 | | 4 | | 4 |  | | 8 | | 8 | | 0 |  | | |  |
| **Grade** | |  | |  | |  | **0.010** | |  | |  | |  | 0.138 | | |  |
|  | Low | 22 | | 18 | | 4 |  | | 23 | | 14 | | 9 |  | | |  |
|  | High | 113 | | 59 | | 54 |  | | 112 | | 85 | | 27 |  | | |  |
| **AJCC stage** | |  |  | | |  | 0.886 | |  | |  | |  | 0.173 | | |  |
|  | II | 83 | 47 | | | 36 |  | | 83 | | 61 | | 22 |  | | |  |
|  | III | 44 | 26 | | | 18 |  | | 44 | | 30 | | 14 |  | | |  |
|  | IV | 8 | 4 | | | 4 |  | | 8 | | 8 | | 0 |  | | |  |
| **ACT** | |  |  | | |  | 0.979 | |  | |  | |  | 0.299 | | |  |
|  | Applied | 65 | 37 | | | 28 |  | | 65 | | 45 | | 20 |  | | |  |
|  | Not applied | 70 | 40 | | | 30 |  | | 70 | | 54 | | 16 |  | | |  |
| **Events** | |  |  | | |  |  |  | | |  | |  |  | | |  |
|  | Death | 70 | 32 | | | 38 | **0.006** | 70 | | | 53 | | 18 | 0.795 | | |  |
|  | Recurrence | 63 | 24 | | | 39 | **0.001** | 63 | | | 50 | | 13 | 0.138 | | |  |
| IQR: interquartile range; AJCC: American Joint Committee on Cancer; LVI: lymphovascular invasion; ACT: adjuvant chemotherapy. | | | | | | | | | | | | | | | |  |  |
| *P** value from Fisher’s exact test was used when data fail to meet the requirement of Chi-square test. | | | | | | | | | | | | | | | |  |  |
| † Student’s t test. | | | | | | | | | | | | | | | |  |  |

| **Supplementary Table 2 Clinicopathological characteristics and relationship with VISTA^+^ ICs infiltration in TCGA cohort.** | | | | | | | |
| --- | --- | --- | --- | --- | --- | --- | --- |
|  | | **TCGA cohort ( *n* = 391 )** | | | | | |
| **Characteristics** | | **Patients** | **VISTA^+^ ICs signature** | |  | |  |
|  |  | **No.** | **Low** | **High** | | ***P**** | |
| **Total** | | 391 | 226 | 165 | |  | |
| **Gender** | |  |  |  | | **0.033** | |
|  | Male | 285 | 174 | 111 | |  | |
|  | Female | 106 | 52 | 54 | |  | |
| **LVI** | |  |  |  | | 0.153 | |
|  | Absent | 125 | 76 | 49 | |  | |
|  | Present | 142 | 74 | 68 | |  | |
| **pT stage** | |  |  |  | | **0.001** | |
|  | pT2 | 113 | 81 | 32 | |  | |
|  | pT3 | 189 | 94 | 95 | |  | |
|  | pT4 | 56 | 25 | 31 | |  | |
| **AJCC stage** | |  |  |  | | **0.001** | |
|  | II | 125 | 89 | 36 | |  | |
|  | III | 137 | 70 | 67 | |  | |
|  | IV | 129 | 67 | 62 | |  | |
| **Events** | |  |  |  | |  | |
|  | Death | 173 | 87 | 86 | | **0.007** | |
| LVI: lymphovascular invasion; AJCC: American Joint Committee on Cancer | | | | | | | |
| *P** value from Fisher’s exact test was used when data fail to meet the requirement of Chi-square test. | | | | | | | |
| † Student’s t test. | | | | | | | |

| **Supplementary Table 3 Clinicopathological characteristics and relationship with VISTA^+^ ICs infiltration in IMvigor210 cohort.** | | | | | | | |
| --- | --- | --- | --- | --- | --- | --- | --- |
|  | | **IMvigor210 cohort ( *n* = 195 )** | | | | | |
| **Characteristics** | | **Patients** | **VISTA^+^ ICs signature** | |  | |  |
|  |  | **No.** | **Low** | **High** | | ***P**** | |
| **Total** | | 195 | 133 | 62 | |  | |
| **Gender** | |  |  |  | | 0.103 | |
|  | Male | 153 | 100 | 53 | |  | |
|  | Female | 42 | 33 | 9 | |  | |
| **Response** | |  |  |  | | 0.078 | |
|  | CR | 15 | 6 | 9 | |  | |
|  | PR  SD  PD | 27  35  91 | 18  23  67 | 9  12  24 | |  | |
| **PD-L1+ IC level** | |  |  |  | | **0.001** | |
|  | IC0 | 49 | 44 | 5 | |  | |
|  | IC1 | 79 | 57 | 22 | |  | |
|  | IC2+ | 66 | 32 | 34 | |  | |
| **Tumor-immune phenotypes** | |  |  |  | | **0.001** | |
|  | Desert | 48 | 43 | 5 | |  | |
|  | Excluded | 73 | 50 | 23 | |  | |
|  | Inflamed | 46 | 20 | 26 | |  | |
| **Tumor mutation burden** | |  |  |  | | **0.041** | |
|  | TMB^lo^ | 88 | 68 | 20 | |  | |
|  | TMB^hi^ | 69 | 43 | 26 | |  | |
| **Events** | |  |  |  | |  | |
|  | Death | 128 | 93 | 35 | | 0.065 | |
| IC, immune cells; CR, complete response; PR, progressive response; SD, stable disease; PD, partial disease | | | | | | | |
| *P** value from Fisher’s exact test was used when data fail to meet the requirement of Chi-square test. | | | | | | | |
| † Student’s t test. | | | | | | | |

| **Supplementary Table 4** **Immunohistochemistry antibodies and quantification** | | | | | | |
| --- | --- | --- | --- | --- | --- | --- |
| **No.** | **Identified cells** | **IHC antibody** | **Clonality Species** | **Company** | **Product No.** | **Diluted** |
| 1 | VISTA^+^ cells | Anti-VISTA antibody | Monoclonal Rabbit Anti-human | Abcam | ab243891 | 1:250 |
| 2 | B cells | Anti-CD19 antibody | Monoclonal Rabbit Anti-human | Abcam | Ab31947 | 1:400 |
| 3 | CD8^+^ T cells | Anti-CD8 alpha antibody | Monoclonal Mouse Anti-human | Abcam | ab17147 | 1:100 |
| 4 | CD4^+^ T cells | Anti-CD4 antibody | Monoclonal Mouse Anti-human | Abcam | ab67001 | 1:50 |
| 5 | Tregs | Anti-FOXP3 antibody | Monoclonal Mouse Anti-human | Abcam | ab22510 | 1:100 |
| 6 | M1 | Anti-CD68 antibody  Anti-HLA-DR antibody | Monoclonal Mouse Anti-human  Monoclonal Rabbit Anti-human | Dako  Abcam | IR604  ab92511 | prediluted  1:250 |
| 7 | M2 | Anti-MRC1 antibody | Monoclonal Rabbit Anti-human | Sigma | HPA004114 | 1:500 |
| 8 | DCs | Anti-CD11c antibody | Monoclonal Rabbit Anti-human | Abcam | ab52632 | 1:500 |
| 9 | Mast cells | Anti-Mast Cell antibody | Monoclonal Mouse Anti-human | Abcam | ab2378 | 1:10000 |
| 10 | Neutrophil | Anti-CD66b antibody | Polyclonal Rabbit Anti-human | Abcam | Ab197678 | 1:1000 |
| 11 | PD-1^+^ cells | Anti-PD1 antibody | Monoclonal Mouse Anti-human | Abcam | ab52587 | 1:100 |
| 12 | PD-L1^+^ cells | Anti-PD-L1 antibody | Monoclonal Rabbit Anti-human | Abcam | ab228415 | 1:500 |
| 13 | CTLA-4^+^ cells | Anti-CTLA-4 antibody | Monoclonal Mouse Anti-human | Santa-Cruz | sc-376016 | 1:100 |
| 14 | TIM-3^+^ cells | Anti-TIM-3 antibody | Monoclonal Rabbit Anti-human | Abcam | ab185703 | 1:100 |
| 15 | LAG-3^+^ cells | Anti-Lymphocyte Activation Gene 3 antibody | Monoclonal Rabbit Anti-human | Abcam | ab209236 | 1:500 |
| 16 | TIGIT^+^ cells | Anti-TIGIT antibody | Monoclonal Rabbit Anti-human | Abcam | ab243903 | 1:100 |
| 17 | IL-10^+^ cells | Anti-IL-10 antibody | Monoclonal Mouse Anti-human | Abcam | ab134742 | 1:200 |
| 18 | TGF-β^+^ cells | Anti-LAP antibody | Polyclone Goat Anti-human | R&D | AB-246-NA | 1:100 |
| 19 | IFN-γ^+^ cells | Anti-Interferon gamma antibody | Monoclonal Rabbit Anti-human | Abcam | ab9657 | 1:300 |
| 20 | GZMB^+^ cells | Anti-Granzyme B antibody | Monoclonal Rabbit Anti-human | Abcam | ab4059 | 1:200 |

VISTA, V domain Ig suppressor of T cell activation; Tregs: regulatory T cells; M1, M1 like Macrophages; M2, M2 like Macrophages; DCs: dendritic cells; PD-1: Programmed cell death protein 1; PD-L1: programmed cell death 1 ligand 1; CTLA-4: Cytotoxic T lymphocyte-associated protein 4; TIM-3: T-cell immunoglobulin mucin-3; LAG-3: lymphocyte activation gene 3; TIGIT: T-cell immunoglobulin and ITIM domain; IL10: interleukin 10; TGF-β: transforming growth factor-β; IFN-γ: interferon-γ; GZMB: granzyme B

**Supplementary Table 5 Specific gene signatures**

| **Signature** | **Genes** |
| --- | --- |
| VISTA^+^ ICs signature infiltration | *VISTA, IL6, IL10, IGSF11, SELPLG, VSIG8, ESAM, CD45* |
| M2 macrophages  proinflammatory | *ABCA9, ABHD1, ACSS1, ACYP2, ADA, AIF1, ALAD, ARFGEF3, ARSB, ATP6V0E2, AXL, BCKDHB, BLOC1S6, CADM1, CAP1, CAPN5, CBX6, CD59, CFH, CLBA1, CNRIP1, COLEC12, COMT, CRIM1, CXCL14, CXCR4, DST, DYNLT1, EMC1, ENO2, FAM124A, FAM135A, FAM9B, FGD2, FILIP1L, GALNT11, GATM, GDA, GJA1, GLO1, GNB4, HAUS2, HDDC3, HLA-DQA1, HMGN3, KCNJ10, LAMA3, LCORL, LYPLAL1, MAF, MALAT1, MARCKSL1, MARCO, MSR1, NAT8L, NRCAM, OCEL1, OGFRL1, P2RY13, PIANP, PIK3AP1, PLAAT3, PLBD1, PLXDC2, PPP2R5C, PTGER3, RAB10, RAPSN, RASAL2, RCBTB2, RCN1, RFX3, RPL14, SFI1, SLC35A1, SLC7A7, SLCO2B1, SRD5A3, TGFBI, TIFAB, TM7SF3, TOR3A, TTC3, TUBB2B, TXNIP, ZNF727* |
| Naïve CD4 T cells transform into Treg cells | *ADA, ADSS2, AGPAT4, AGT, AK7, ALG8, AOPEP, AQP8, ARID4B, ARL5A, ATP2A2, ATP2B4, AVEN, BCL3, BUB1B, C17orf98, CALR, CAPRIN1, CASTOR1, CCS, CDC14B, CEP57L1, CHCHD10, CLSPN, CNDP2, CNTROB, COL15A1, CRIP1, CSRP2, CUBN, CYP2D6, DAG1, DBNDD2, DDN, DGAT1, DLGAP3, DNAAF4, DNAAF5, DUSP4, E2F7, ECM2, ENTPD1, EPSTI1, ETF1, EXOC2, EXOSC8, F2RL3, FAF1, FAM72A, FAM81A, FAM83D, FNTB, FOXC1, FOXP3, GALK1, GBP4, GBP7, GCAT, GM2A, GPR83, GRB7, GZMB, H2AZ1, HBS1L, HEMK1, HIF1A, HINT1, HIPK2, HMGCR, HNRNPAB, HSPD1, IFT57, IFT80, IGHM, IGKC, IKZF2, IKZF4, IL17RB, IL1RL1, IL2RA, IL2RB, IL4R, IMPDH2, IRAK1BP1, ITGA6, ITGAE, ITIH5, KCNJ15, KLC3, KPNA2, KRAS, LANCL2, LARP1, LCA5, LCLAT1, LGALS7, LINC00167, LSR, MAN1A1, MEDAG, MELK, MIF, MIF4GD, MKLN1, MLST8, MTHFD1L, MUC1, MYB, N4BP2, NABP1, NCOA3, NDRG1, NEB, NHP2, NHSL2, NIBAN1, NIBAN3, NINJ2, NME1, NPC1, NUCB2, NXPE4, P4HA1, PARD6G, PATL2, PDCD1LG2, PDE2A, PDLIM4, PENK, PGPEP1L, PHLPP1, PIK3R3, PIWIL2, PLAGL1, PPA1, PPM1J, PRNP, PTAFR, PTGR1, PTP4A3, RAB26, RANBP1, RAPSN, RBBP7, RBM38, RCSD1, REEP5, REXO2, RIMKLA, RNF39, RRAGD, RRAS2, RWDD1, S100A6, SAMHD1, SCD, SEC24A, SELL, SESN1, SGO2, SHMT2, SLC2A3, SLC30A2, SLC35D1, SNHG11, SOCS2, SRGN, SRSF2, STIM2, STON1, STX1A, SYPL1, TBCA, TBXA2R, TDGF1, TEC, TENT5A, TGFBR1, TLE2, TM4SF5, TMEM64, TMPRSS4, TNFRSF18, TNFRSF4, TNFRSF9, TP53BP2, TP53I11, TPP2, TREML1, TREML2, TRIM59, TRIP13, TUBE1, UBE2D2, VAV2, XBP1, XKR5, YWHAZ, ZBTB18, ZBTB32*, |
| Exhausted CD8^+^ T cells | *ACOX3, AF131762, AI718865, AI918632, AK022346, AK022390, AK074696, AK093729, AK093729, AK093194, AK098360, AK130583, AK2, AKR1A1, AL133570, ALG10B, ANGPTL6, APEH, ATF3, ATF3, ATF4, AW361634, BC038559, BC041926, BE008305, BE739632, BECN1, BF088423, BF475893, BG190831, BG950086, BQ002790, BST2, BX095032, BX360933, C10orf26, C13orf15, C13orf15, C19orf56, C6orf114, CA866957, CALML4, CBLB, CCT6A, CD27, CD619445, CKLF, CLTB, COTL1, CPT1C, CRTAM, CSNK2A2, CTLA4, CXCL13, DDX11, DGKE, CTNBP1, DUSP16, DUSP4, DUSP5, ERBB2IP, ERF, EXOC4, EXOC5, EXOC7, FAM105B, FAM53C, FASLG, FCRL3, FLJ20160, FOS, GALM, GAPVD1, GARNL1, GBP1, GCC2, GEM, HSPD1, ICOS, IFNG, ILF2, IRF4, ITGA1, ITM2A, JMJD1C, JTB, KIAA0319L, KIAA1627, KIF3B, LASP1, LIX1L, LOC148413, LOC220594, LOC284454, LOC442421, LOC643894, LOC644422, LOC647907, LOC651147, MAPRE2, MBNL1, MCL1, MCM6, MYO7A, NDFIP1, NDUFC2, NEIL2, NFAT5, NOD2, NPLOC4, OTUD7B, PARVB, PARVG, PBEF1, PCBP2, PES1, PHLDA1, PIK3IP1, PKM2, PPP1CC, PPP1R2, PRDX5, PSENEN, PTPRJ, RAB27A, RALA, RANBP2, RDH10, RGPD1, RGS1, RGS3, RNF24, RP5-821d11.2, S100PBP, SARDH, SAT1, SECTM1, SEMA7A, SIRPG, SLC7A5, SNF1LK, SNX9, SOD1, SPG7, SPRY1, STAT3, STX1A, TAP1, TATDN1, TBC1D3, TCEAL3, TFG, THC2510958, THC2576044, THC2603416, THC2657597, TMCC1, TNFRSF9, TOPORS, TOX, TP53INP1, TP53INP2, TRAF3, TRAF5, TRBV5-4, TRIB1, TSPYL2, TUBA4A, UBE1L, VTA1, XCL2, YPEL5, ZC3H12A, ZC3H12C, ZFAND5* |

**Supplementary Table 6** **Univariate analysis of clinicopathologic features and VISTA^+^ ICs/ VISTA^+^ TCs infiltration** **in ZSHS Cohort**

| **­ Characteristics** | | **Cox regression analyses ( *n* = 135 )** | |  |
| --- | --- | --- | --- | --- |
|  | | **HR (95%CI)** | ***P**** | |
| **Overall survival** | | | | |
| Gender (male vs. female) | 0.606 (0.322-1.139) | | 0.120 | |
| LVI (present vs. absent) | 1.712 (1.024-2.861) | | **0.040** | |
| pT (3 vs. 2)  (4 vs. 2) | 1.317 (0.727-2.387)  1.700 (0.939-3.079) | | 0.364  0.080 | |
| AJCC (III vs. II)  (IV vs. II) | 1.437 (0872-2.368)  2.628(1.104-6.252) | | 0.155  **0.029** | |
| VISTA^+^ ICs infiltration  (high vs. low) | 2.388 (1.462-3.900) | | **0.001** | |
| VISTA^+^ TCs infiltration  (positive vs. negative) | 1.056 (0.617-1.806) | | 0.843 | |
| **Recurrence-free survival** | | | | |
| Gender (male vs. female) | 0.670 (0.357-1.258) | | 0.213 | |
| LVI (present vs. absent) | 1.361 (0.806-2.300) | | 0.249 | |
| pT (3 vs. 2)  (4 vs. 2) | 1.482 (0.811-2.709)  1.850 (0.962-3.559) | | 0.201  0.065 | |
| AJCC (III vs. II)  (IV vs. II) | 1.480 (0.865-2.531)  3.267(1.438-7.420) | | 0.153  **0.005** | |
| VISTA^+^ ICs infiltration  (high vs. low) | 3.174 (1.896-5.313) | | **0.001** | |
| VISTA^+^ TCs infiltration  (positive vs. negative) | 0.760 (0.412-1.400) | | 0.378 | |

HR, Hazard Ratio; CI, confidence interval; LVI, lymphovascular invasion; AJCC, American Joint Committee on Cancer

Significant *P* value was shown in bold

**Supplementary Table 7 Univariate analysis of clinicopathologic features and VISTA^+^ ICs signature infiltration in TCGA Cohort**

| **­ Characteristics** | | **Cox regression analyses ( *n* = 135 )** | |  |
| --- | --- | --- | --- | --- |
|  | | **HR (95%CI)** | ***P**** | |
| **Overall survival** | | | | |
| Gender (male vs. female) | 0.884 (0.637-1.226) | | 0.459 | |
| LVI (present vs. absent) | 2.269 (1.552-3.317) | | **0.001** | |
| pT (3 vs. 2)  (4 vs. 2) | 1.838 (1.242-2.721)  2.813 (1.733-4.565) | | **0.002**  **0.001** | |
| AJCC (III vs. II)  (IV vs. II) | 1.646 (1.075-2.520)  2.870 (1.923-4.282) | | **0.022**  **0.001** | |
| VISTA^+^ ICs signature infiltration  (high vs. low) | 2.388 (1.462-3.900) | | **0.001** | |

HR, Hazard Ratio; CI, confidence interval; LVI, lymphovascular invasion; AJCC, American Joint Committee on Cancer

Significant *P* value was shown in bold

**Supplementary Table 8 Multivariate analysis of clinicopathologic features and VISTA^+^ TCs infiltration in ZSHS Cohort**

| **­ Characteristics** | | **Cox regression analyses ( *n* = 135 )** | |  |
| --- | --- | --- | --- | --- |
|  | | **HR (95%CI)** | ***P**** | |
| **Overall survival** | | | | |
| Gender (male vs. female) | 0.561 (0.291-1.081) | | 0.084 | |
| LVI (present vs. absent) | 1.525 (0.881-2.640) | | 0.131 | |
| pT (3 vs. 2)  (4 vs. 2) | 0.628 (0.106-3.699)  0.889 (0.144-5.483) | | 0.607  0.899 | |
| AJCC (III vs. II)  (IV vs. II) | 1.846 (0.302-11.298)  3.387 (0.784-14.631) | | 0.507  0.102 | |
| VISTA^+^ TCs infiltration  (positive vs. negative) | 1.038 (0.597-1.804) | | 0.896 | |
| **Recurrence-free survival** | | | | |
| Gender (male vs. female) | 0.575 (0.297-1.110) | | 0.099 | |
| LVI (present vs. absent) | 1.146 (0.648-2.026) | | 0.948 | |
| pT (3 vs. 2)  (4 vs. 2) | 1.505 (0.272-8.311)  2.221 (0.372-13.267) | | 0.639  0.382 | |
| AJCC (III vs. II)  (IV vs. II) | 0.834 (0.144-4.845)  2.392 (0.559-10.225) | | 0.840  0.239 | |
| VISTA^+^ TCs infiltration  (positive vs. negative) | 0.821 (0.438-1.541) | | 0.540 | |

HR, Hazard Ratio; CI, confidence interval; LVI, lymphovascular invasion; AJCC, American Joint Committee on Cancer

Significant *P* value was shown in bold
